# Commentary: *Piscirickettsia salmonis* Produces a N-Acetyl-L-Homoserine Lactone as a Bacterial Quorum Sensing System-Related Molecule

**DOI:** 10.3389/fcimb.2022.858387

**Published:** 2022-03-04

**Authors:** Héctor A. Levipan, Luis Reyes-Garcia, Ruben Avendaño-Herrera

**Affiliations:** ^1^ Laboratorio de Ecopatología y Nanobiomateriales, Departamento de Ciencias y Geografía, Facultad de Ciencias Naturales y Exactas, Universidad de Playa Ancha, Valparaíso, Chile; ^2^ Departamento de Ciencias Básicas, Facultad de Ciencias, Universidad Santo Tomás, Viña del Mar, Chile; ^3^ Laboratorio de Patología de Organismos Acuáticos y Biotecnología Acuícola, Facultad de Ciencias de la Vida, Universidad Andrés Bello, Viña del Mar, Chile; ^4^ Interdisciplinary Center for Aquaculture Research (INCAR), Universidad Andrés Bello, Viña del Mar, Chile; ^5^ Centro de Investigación Marina Quintay (CIMARQ), Universidad Andrés Bello, Quintay, Chile

**Keywords:** *Piscirickettsia salmonis*, SRS, Piscirickettsiosis, quorum sensing (QS), N-acetyl-L-homoserine Lactone, N-acyl-homoserine lactone (AHL)

Ruiz et al. (2021) recently published a study titled “*Piscirickettsia salmonis* Produces a N-Acetyl-L-Homoserine Lactone as a Bacterial Quorum Sensing System-Related Molecule” in Frontiers in Cellular and Infection Microbiology. The title and the summary of the respective article state to adduce evidence on the unambiguous production of an AHL-type molecule by *P. salmonis*, specifically, the N-acetyl-L-homoserine lactone or C2-HSL (C_6_H_9_NO_3_). The evidence came from two sources, one using a cep-based fluorescent biosensor plasmid harboured in *Pseudomonas putida* EL106 (RPL4cep) and the other using gas chromatography-tandem mass spectrometry (GC-MS). The application of biosensors with different detection thresholds (e.g., fluorescence- versus colorimetric-based biosensors) has been widely reported for the detection of AHL-type molecules. For instance, colorimetric-based biosensors, such as *Chromobacterium violaceum*, can even display a range of limits of detection for AHLs (e.g., 1-1000 nM) depending on the assayed molecule (Girard et al., 2017). However, biosensors ultimately represent just preliminary screening tests for potential quorum-sensing cues. Indeed, bacteriological biosensors require further procedures for more reliable and comprehensive detection and identification of AHLs, such as GC-MS analyses. In a previous study authored by our group, we did not find evidence supporting the existence of a functional QS system based on the endogenous production of AHL-type molecules in two strains of *P. salmonis* by using *C*. *violaceum*-based biosensors coupled with GC-MS (Levipan et al., 2021), including the type strain *P. salmonis* LF-89^T^ (i.e., the same type strain analysed by Ruiz and colleagues).

The sources of evidence provided by Ruiz et al. (2021) seem to supply complementary results; if so, is enough robust GC-MS evidence provided by the authors to prove C2-HSL production? Our primary concerns regarding the main finding reported by Ruiz and colleagues precisely refer to this point. Even though a same retention time was reported by Ruiz and colleagues for the compound of interest (seemingly C2-HSL) using organic extracts from culture supernatants of the two tested *P*. *salmonis* strains (i.e., peak selective ion monitoring [SIM] at 10.459 min), the mass spectrum m/z for extracts from *P. salmonis* LF-89^T^ do not match those generated from *P. salmonis* Ps007 extracts. For instance, fragments m/z 112 and 113 were found almost in the same proportion in the LF-89^T^ spectrum, while in the Ps007 spectrum, these were quite different from the 1:1 ratio (see Figure 4 “Identification of AHLs molecules in *P. salmonis* culture organic extract by GC/MS analysis” in Ruiz et al., 2021; other examples are included in the same figure). These differences could be due to improper noise elimination or to the presence of different compounds in the LF-89^T^ extracts versus the Ps007 extracts. Moreover, the reported m/z 112 and 113 peaks were not explained or supported by references as characteristic ion fragments associated with mass spectra patterns from bacterial AHL extracts.

Ruiz et al. (2021) argued that fragmentation patterns in scan mode exhibited two typical signatures (m/z 57 and 143) for the identification of AHLs in both *P. salmonis* extracts (Cataldi et al., 2004), in addition to the fragments at m/z = 43 and 83 and other m/z values of AHLs that were distinct from C2-HSL (see Table 1 “Experimental retention times and *m/z* fragments detected for standards AHLs molecules and samples analyzed” in Ruiz et al., 2021). One of the few studies suggesting the bacterial production of C2-HSL as a putative AHL involved in quorum sensing (Liu et al., 2019) reported a fragment ion equal to 102 using liquid chromatography-tandem mass spectrometry. Given the current lack of GC-MS data for the compound of interest, the inclusion of liquid chromatography-tandem mass spectrometry analyses for *P. salmonis* extracts could have been useful for comparative purposes. On the other hand, even though the ion at m/z 143 (resulting from a McLafferty rearrangement) is commonly used as a marker fragment for the screening of AHLs when the mass detector is operated in the SIM mode (Cataldi et al., 2004), this ion may also be potentially used as marker for the identification of other bacterial metabolites (Kaal et al., 2009; Elsakhawy et al., 2019).

We agree with Ruiz and colleagues that the m/z fragments detected in the *P. salmonis* extracts match around 80% with the m/z fragments detected for acyl-HSL standards registered in the National Institute of Standards and Technology (NIST) database. However, taking into account the aspects discussed herein, the fact that C2-HSL has not yet been isolated from bacterial extracts, and the lack of mass spectra for pure C2-HSL in the NIST database for comparison purposes, we believe that the results reported by Ruiz et al. (2021) should have been interpreted with major caution, especially since no C2-HSL standard was included in the study. A standard (synthetized at the laboratory or commercially available) would have been useful to check the retention time (at 10.459 min) and the mass spectrum. Therefore, even though the reported retention time is plausible and there is a similarity between the mass spectra of the unknown compound of interest to those from AHLs of longer-side chains, it is important keep in mind that plausibility and similarity are not enough criteria to guarantee and propose rigorous identification for any chemical substance.

In general, the identification of AHLs in culture media is a tricky issue and should be evaluated carefully before the production of a particular AHL by some bacterial strain can be claimed. For example, the evaluation of the response of the biosensors to non-inoculated culture media and non-inoculated and extracted culture media is very important. These negative controls allow to discard that the studied molecule is *a priori* present in a fresh culture medium and hence an eventual background activation of the biosensor (e.g., *P*. *putida*) not associated to the assayed bacterium (*P*. *salmonis* in this case). The AUSTRAL-SRS broth is a complex medium so that the presence of compounds able to modulate quorum-sensing systems based on LuxR transcriptional activator cannot be discarded. For example, we have detected the presence of diketopiperazine-like molecules (Holden et al., 1999) in extracts obtained from relatively simple, autoclaved, and non-inoculated culture media (unpublished data).

Scientific knowledge regarding molecular signalling pathways to control cell density-dependent phenotypes in *P. salmonis* is still in its infancy. A rapid advance in this field will rely on the falsifiability of hypotheses founded on the accumulation of rigorous scientific evidence. We believe that the results reported by Ruiz and colleagues will contribute directly or indirectly to this end; however, until future confirmation of unmistakable C2-HSL production by the aforementioned and other strains of *P. salmonis*, and the elucidation of its role as a quorum-sensing cue in this bacterium, this report should be taken with some caution.

## Author Contributions

HAL and RA-H wrote the manuscript with the support from LR-G. The original idea was conceived by HAL and RA-H. All authors contributed to the article and approved the submitted version.

## Funding

This work was funded by Grants FONDAP-INCAR Center 15110027, PAI/Convocatoria Nacional Subvención a la Instalación en la Academia, Convocatoria 2018 Folio 77180039 and FONDECYT Iniciación 11200708 from the Agencia Nacional de Investigación y Desarrollo (ANID, Chile).

## Conflict of Interest

The authors declare that the research was conducted in the absence of any commercial or financial relationships that could be construed as a potential conflict of interest.

## Publisher’s Note

All claims expressed in this article are solely those of the authors and do not necessarily represent those of their affiliated organizations, or those of the publisher, the editors and the reviewers. Any product that may be evaluated in this article, or claim that may be made by its manufacturer, is not guaranteed or endorsed by the publisher.
